# Magnetic and Electrical Behaviors of the Homo- and Heterometallic 1D and 3D Coordination Polymers Based on the Partial Decomposition of the [Cr(C_2_O_4_)_3_]^3−^ Building Block

**DOI:** 10.3390/ma13235341

**Published:** 2020-11-25

**Authors:** Lidija Kanižaj, Pavla Šenjug, Damir Pajić, Luka Pavić, Krešimir Molčanov, Marijana Jurić

**Affiliations:** 1 Ruđer Bošković Institute, Bijenička cesta 54, 10000 Zagreb, Croatia; Lidija.Kanizaj@irb.hr (L.K.); lpavic@irb.hr (L.P.); Kresimir.Molcanov@irb.hr (K.M.); 2 Department of Physics, Faculty of Science, University of Zagreb, Bijenička cesta 32, 10000 Zagreb, Croatia; psenjug@phy.hr (P.Š.); dpajic@phy.hr (D.P.)

**Keywords:** coordination polymers, oxalate-bridged, crystal structure, electrical property, magnetic property

## Abstract

One-dimensional (1D) oxalate-bridged homometallic {[Mn(bpy)(C_2_O_4_)]·1.5H_2_O}*_n_* (**1**) (bpy = 2,2’-bipyridine) and heterodimetallic {[CrCu_3_(bpy)_3_(CH_3_OH)(H_2_O)(C_2_O_4_)_4_][Cu(bpy)Cr(C_2_O_4_)_3_]·CH_2_Cl_2_·CH_3_OH·H_2_O}*_n_* (**2**) coordination polymers, as well as the three-dimensional (3D) heterotrimetallic {[CaCr_2_Cu_2_(phen)_4_(C_2_O_4_)_6_]·4CH_3_CN·2H_2_O}*_n_* (**3**) (1,10-phenanthroline) network, have been synthesized by a building block approach using a layering technique, and characterized by single-crystal X-ray diffraction, infrared (IR) and impedance spectroscopies and magnetization measurements. During the crystallization process partial decomposition of the tris(oxalato)chromate(III) happened and 1D polymers **1** and **2** were formed. The antiferromagnetic interactions between the manganese(II) ions were mediated by oxalate ligands in the chain [Mn(bpy)(C_2_O_4_)]*_n_* of **1**, with intra-chain super-exchange interaction 𝐽 = (−3.134 ± 0.004) K; magnetic interaction between neighbouring chains is negligible making this system closer than other known Mn-chains to the ideal 1D Heisenberg antiferromagnet. Compound **2** comprises a 1D coordination anion [Cu(bpy)Cr(C_2_O_4_)_3_]*_n_^n^*^−^ (**Cr2–Cu4**) with alternating [Cr(C_2_O_4_)_3_]^3^*^−^* and [Cu(bpy)]^2+^ units mutually bridged through the oxalate group. Another chain (**Cr1–Cu3**) is similar, but involves a homodinuclear unit [Cu(bpy)(H_2_O)(*µ*-C_2_O_4_)Cu(bpy)(CH_3_OH)]^2+^ (**Cu1–Cu2**) coordinated as a pendant group to a terminal oxalate oxygen. Magnetic measurements showed that the **Cu1−Cu2** cationic unit is a strongly coupled antiferromagnetic dimer, independent from the other magnetic ions within ferromagnetic chains **Cr1–Cu3** and **Cr2–Cu4**. A 3D polymer {[CaCr_2_Cu_2_(phen)_4_(C_2_O_4_)_6_]·4CH_3_CN·2H_2_O}*_n_* (**3**) comprising three different metal centers (Ca^2+^, Cr^3+^ and Cu^2+^) oxalate-bridged, contains Ca^2+^ atoms as nodes connected with four Cr^3+^ atoms through oxalate ligands. The network thus formed can be reduced to an underlying graph of diamondoid (**dia**) or (6^6^) topology. Magnetization of **3** shows the ferromagnetic oxalate-bridged dimers [Cu^II^Cr^III^], whose mutual interaction could possibly originate through the spin polarization of Ca^2+^ orbitals. Compounds **1** and **3** exhibit lower electrical conductivity at room temperature (RT) in comparison to compound **2**.

## 1. Introduction

The design and synthesis of new materials with targeted physical properties represents an operative area of research for materials scientists, chemists and physicists. Metal-organic coordination polymers due to structural diversity allow the introduction of two (or even more) properties leading to multifunctional materials. By combining the intrinsic properties of the host, especially the magnetic ones, with those of the selected guest molecules, upgrading and expanding the molecular magnetism toward multifunctional compounds has been achieved [1,2,3].

The oxalate group has been used as one of the most versatile ligands for preparation of these types of material. Its various possibilities of coordination to the metal centres and the ability to mediate magnetic interactions between paramagnetic metal ions has enabled synthesis and characterization of a large number of oxalate-based transition-metal species of different nuclearity and dimensionality, many of them having tunable magnetic frameworks [4]. An appropriate approach for creating hybrid magnetic materials including multifunctional properties is the combination of magnetic oxalate-based coordination polymers with organic/inorganic functional cations. Most of the oxalate-based molecular magnets described so far have been obtained by the “complex-as-ligand approach” [5]. This means that the tris(oxalato)metalate [M^III^(C_2_O_4_)_3_]^3−^ anion (M^III^ = Cr, Mn, Fe, Ru, Rh, or V) is used as a molecular building block, as a ligand towards another metal cations. A templating counterion controls the topology of these oxalate-bridged species. Therefore, layered two-dimensional (2D) honeycomb structures of the formulae [M^II^_2_(C_2_O_4_)_3_]*_n_^2n−^* and [M^II^M^III^(C_2_O_4_)_3_]*_n_^n−^*, showing ferro-, ferri- or canted antiferromagnetic ordering, have been obtained using a bulky charge-compensating molecular cation [6,7,8,9,10,11]. For example, a molecular ferromagnetic conductor as a multifunctional material was formed by introducing bis(ethylenedithio)tetrathiafulvalene (BEDT-TTF) cationic stacks, showing electron conduction due to the organic π-electron donor, between honeycomb anionic layers [MnCr(C_2_O_4_)_3_]*_n_^n−^* [12]. Magnetic networks which are different from the 2D honeycomb-like network can also be formed depending on the nature of the templating cation (size, shape, and charge). Using the tris-chelated [M(bpy)_3_]^2+^ or [M(bpy)_3_]^3+^ (bpy = 2,2′-bipyridine) entities the family of 3D networks of the formulae [M^II^_2_(C_2_O_4_)_3_]*_n_*^2*n*−^, [M^I^M^III^(C_2_O_4_)_3_]*_n_*^2*n*−^, and [M^II^M^III^(C_2_O_4_)_3_]*_n_^n−^* have been prepared [1,4,13,14,15]. The introduction of the capping ligands, as organic ones containing *N*-donors, in addition to the stabilization of the solid-state structures, can control and influence the dimensionality of coordination systems [1].

Recently, proton conductivity is a new performance of the coordination polymers since they could provide required proton-conducting pathways, mostly including water as conducting media. Also, proton conductivity has a need of proton carriers like H_3_O^+^, NH_4_^+^, or H^+^ belonging to acid groups or to networks of hydrogen bonds. Practical proton conductors should work under ambient conditions to avoid desorption of the water molecules from these materials under low humidity due to their low affinity. It is known that in the metal-organic systems the hydrophilic interaction can firmly influence the affinity to water molecules showing good proton conductivity even under ambient conditions (i.e., low humidity and low temperatures). In general, the number of papers related to proton conductivity of coordination polymers is still small [16,17,18,19]. One of them describes a 3D oxalate-based polymer {(NH_4_)_5_[Mn_2_Cr_3_(C_2_O_4_)_9_]·10H_2_O}*_n_*, to which an anion network provides antiferomagnetic long-range ordering while cations cause high humidity-dependent proton conductivity [20]. For the same reason, oxalate-bridged bimetallic systems {[NH(prol)_3_][MCr(C_2_O_4_)_3_]}*_n_* (M = Mn^2+^, Fe^2+^, Co^2+^; NH(prol)_3_^+^ = tri(3-hydroxypropyl)ammonium) exhibit the coexistence of technologically useful behavior such as ferromagnetism and proton conductivity [21], similar to the metal-organic quartz-like framework {(NH_4_)_4_[MnCr_2_(C_2_O_4_)_6_]·4H_2_O}*_n_* [22].

In this work, as a continuation of our magneto-structural studies about the oxalate-bridged polymers prepared by a building block approach [23,24,25,26,27], we report crystal structures, electrical and magnetic properties of novel oxalate-bridged coordination compounds: one-dimensional (1D) homometallic {[Mn(bpy)(C_2_O_4_)]·1.5H_2_O}*_n_* (**1**) and heterodimetallic {[CrCu_3_(bpy)_3_(CH_3_OH)(H_2_O)(C_2_O_4_)_4_][Cu(bpy)Cr(C_2_O_4_)_3_]·CH_2_Cl_2_·CH_3_OH·H_2_O}*_n_* (**2**) polymers, and a three-dimensional (3D) heterotrimetallic {[CaCr_2_Cu_2_(phen)_4_(C_2_O_4_)_6_]·4CH_3_CN·2H_2_O}*_n_* (**3**) (1,10-phenanthroline) network. Structural diversity reflected on magnetic and electrical properties of these polymers, containing bpy or phen as an additional ligand, all prepared using same precursor [Cr(C_2_O_4_)_3_]^3−^, has been investigated by single-crystal X-ray diffraction, infrared (IR) and impedance spectroscopies and magnetization measurements. Interestingly, compounds **1** and **2** have been obtained as a consequence of the partial decomposition of the used building block [5]. Also, for the first time, polymer **2** possessed a 1D arrangement of copper(II) and chromium(III) ions mutually bridged by the bis(bidentate) oxalate group, while **3** is the first structurally known oxalate-based compound containing these three metal ions [4].

## 2. Experiment

### 2.1. Materials and Methods

All used chemicals were procured from commercial sources and used without further purification. The starting precursor K_3_[Cr(C_2_O_4_)_3_]∙3H_2_O was synthesized according to the literature method [28]. Elemental analyses for C, H and N were performed with a microanalytical analyzer Perkin–Elmer Model 2400. The infrared spectra were recorded using KBr pellets by a Bruker Alpha-T spectrometer in the 4000–350 cm^−1^ range. Thermal properties were investigated from room temperature (RT) to 1000 °C in a nitrogen atmosphere with a Shimadzu DTG-60H analyzer (heating rate of 10 °C min^−^^1^).

### 2.2. Synthetic Procedures

#### 2.2.1. Synthesis of {[Mn(bpy)(C_2_O_4_)]·1.5H_2_O}_*n*_ (**1**)

An aqueous solution (4 mL) of K_3_[Cr(C_2_O_4_)_3_]∙3H_2_O (0.049 g; 0.1 mmol) was layered with a mixture of the methanol solutions containing bpy (0.016 g; 0.1 mmol; 4 mL) and MnCl_2_·4H_2_O (0.020 g; 0.1 mmol; 4 mL) in a test tube. After two weeks, yellow crystals of **1** were formed, isolated and washed with a small amount of water and dried in air. The yield was 51%. When the test tube was left open, and the reaction mixture evaporated, the black powder of the starting chromium(III) precursor was obtained (confirmed by IR spectroscopy). Analytical calculation (Anal. Calc.) for C_12_H_9_MnN_2_O_5_ (**1**; *M*_r_ = 324.15): C, 44.46; H, 2.80; N, 8.64: Found. C, 44.61; H, 2.86; N, 8.87%. IR spectroscopy data (KBr, cm^−1^): 3436 (m), 3089 (w), 3026 (w), 2927 (w), 1673 (vs), 1611 (vs), 1574 (sh), 1488 (w), 1472 (m), 1443 (m), 1383 (w), 1362 (w), 1310 (m), 1244 (w), 1176 (w), 1162 (w), 1113 (w), 1057 (w), 1040 (w), 1016 (m), 1009 (sh), 793 (m), 769 (m), 737 (w), 650 (w), 627 (w), 541 (w), 492 (w), 410 (w).

#### 2.2.2. Synthesis of {[CrCu_3_(bpy)_3_(CH_3_OH)(H_2_O)(C_2_O_4_)_4_][Cu(bpy)Cr(C_2_O_4_)_3_]·CH_2_Cl_2_·CH_3_OH·H_2_O}_*n*_ (**2**)

A methanol/dichloromethane (1:1) mixture solution (8 mL) of CuCl_2_·2H_2_O (0.017 g; 0.1 mmol) and bpy (0.016 g; 0.1 mmol) was layered with an aqueous solution (3 mL) of K_3_[Cr(C_2_O_4_)_3_]∙3H_2_O (0.049 g; 0.1 mmol). After a few days blue crystals of the known homometallic coordination polymer {[Cu(bpy)(C_2_O_4_)]·2.5H_2_O}*_n_* were formed [29], but very soon they decomposed. Green prismatic crystals of compound **2** were obtained after 10 days, isolated and washed with water and dried in air. The yield was 31%. Anal. Calc. for C_57_H_46_Cl_2_Cr_2_Cu_4_N_8_O_32_ (**2;** *M*_r_ = 1784.12): C, 38.37; H, 2.60; N, 6.28: Found. C, 37.99; H, 2.52; N, 6.35%. IR spectroscopy data (KBr, cm^−1^): 3444 (m) 1702 (s), 1679 (vs), 1664 (vs), 1634 (s), 1601 (vs), 1572 (sh), 1495 (w), 1472 (w), 1448 (m), 1430 (m), 1414 (m), 1389 (m), 1352 (w), 1315 (w), 1293 (m), 1274 (sh), 1252 (w), 1174 (w), 1159 (w), 1107 (w), 1057 (w), 1035 (w), 1021 (w), 1005 (w), 905 (w), 814 (m), 804 (m), 773 (m), 730 (m), 700 (w), 664 (w), 650 (w), 641 (w), 609 (sh), 592 (w), 559 (w), 542 (m), 474 (m), 444 (w), 414 (m).

#### 2.2.3. Synthesis of {[CaCr_2_Cu_2_(phen)_4_(C_2_O_4_)_6_]·4CH_3_CN·2H_2_O}_*n*_ (**3**)

An aqueous solution (4 mL) of K_3_[Cr(C_2_O_4_)_3_]∙3H_2_O (0.049 g; 0.1 mmol) was layered with an acetonitrile solution (4 mL) of phen (0.020 g; 0.1 mmol). Then, an acetonitrile solution (5 mL) containing Cu(NO_3_)_2_·3H_2_O (0.025 g; 0.1 mmol) and Ca(NO_3_)_2_·4H_2_O (0.007 g; 0.03 mmol) was carefully laid above the existing layers into a test tube. The dark green prismatic crystals of **3** were obtained after a few days, isolated and washed with a diluted matrix and a small amount of methanol and quickly dried in air. The yield was 63%. Anal. Calc. For C_68_H_48_CaCr_2_Cu_2_N_12_O_26_ (**3**; *M*_r_ = 1720.36): C, 47.47; H, 2.81; N, 9.77: Found. C, 47.48; H, 2.83; N, 9.75%. IR spectroscopy data (KBr, cm^−1^): 3556 (m), 3487 (m), 3063 (w), 2925 (w), 2851 (w), 2369 (w), 2295 (w), 2256 (w), 2249 (w), 1710 (m), 1664 (vs), 1638 (vs), 1589 (sh), 1520 (m), 1496 (w), 1447 (m), 1426 (s), 1416 (s), 1375 (w), 1343 (w), 1282 (m), 1216 (w), 1149 (w), 1108 (w), 1090 (w), 1038 (w), 904 (m), 870 (w), 851 (m), 812 (m), 799 (sh), 781 (w), 725 (m), 647 (w), 543 (m), 507 (w), 476 (m), 419 (m). 

### 2.3. Single-Crystal X-ray Structural Study

The XRD analysis data for single crystals of compounds **1**–**3** were collected by ω-scans on an Oxford Diffraction Xcalibur Nova R diffractometer with mirror-monochromated Cu-Kα radiation (λ = 1.54179 Å, microfocus tube, CCD detector), at 293(2) K (**1** and **3**) and 100(2) K (**2**). Friedel pairs were measured to unambiguously establish absolute structure of compound **3**. Table 1 contains the crystal data and details of data collections and refinements for the structures **1**–**3**. Data reduction and multi-scan absorption correction were made by program package CrysAlis PRO [30]. The structures were solved using SHELXS97 [31] and refined with SHELXL-2017 [32]. The full-matrix least squares refinement was used to refine the models; all non-hydrogen atoms were refined anisotropically. Hydrogen atoms bound to C atoms were modelled as riding entities (the AFIX command in SHELXL-2017 [32]), while hydrogen atoms of water molecules were located in difference Fourier maps and refined with O–H bond length restrained to 0.92(2) Å and HOH angle were restrained by restraining intramolecular H···H distance to 1.50(4) Å.

Molecular geometry calculations were performed by PLATON [33,34] and molecular graphics were prepared using ORTEP-3 [35], and Mercury [36]. Analysis of topology was performed by the program package TOPOS PRO [37]. 

CCDC 2036059–2036061 contains the supplementary crystallographic data for this paper. These data can be obtained free of charge via http://www.ccdc.cam.ac.uk/conts/retrieving.html (or from the CCDC, 12 Union Road, Cambridge CB2 1EZ, UK; Fax: +44 1223 336033; E-mail: deposit@ccdc.cam.ac.uk).

### 2.4. Magnetic Study

Magnetic properties were investigated on polycrystalline powder samples of **1–3** using a MPMS 5 commercial superconducting quantum interferometer device (SQUID) magnetometer (Quantum Design, San Diego, CA, USA). The measured data were corrected for signal of the sample holder/ampoule. The temperature and field dependence of magnetization were measured in the temperature range of 2–300 K, and in the fields up to 5 T. For each value of applied magnetic field *H* temperature dependence of magnetization *M*(*T*) was measured two times while heating, first after the sample was cooled in the zero field (ZFC curve), and the second time after the sample was cooled in the same field in which the *M*(*T*) is measured (FC curve). Magnetic hysteresis *M*(*H*) was measured at several stable temperatures in magnetic fields up to 5 T. For calculation of the molar magnetic susceptibility *χ*, measurement of *M*(*T*) in field of 1000 Oe was used, since it is high enough to reduce the influence of noise and at the same time the *M*(*H*) response is surely linear even well above this field, making the calculated *χ*(*T*) as a reliable physical function. The parameters of magnetic interactions, including exchange, zero field splitting, and *g*-factors, were obtained by appropriate modelling and fitting.

### 2.5. Electrical Study

The electrical properties of compounds **1**–**3** were studied by impedance spectroscopy (IS). An impedance analyzer (Novocontrol Alpha-AN Dielectric Spectrometer, Novocontrol Technologies GmbH & Co. KG, Hundsangen, Germany) was used for measuring complex impedance at room temperature (RT) in the frequency range from 0.1 Hz to 1 MHz. The measurements were performed on polycrystalline samples pressed into pellets of approximate thickness from 0.4 to 0.6 mm and placed between brass electrodes which served as electrical contacts. The experimental data were analyzed by equivalent circuit modelling using the complex non-linear least-squares (CNLLSQ) fitting procedure and WinFit software (Version 3.2, Novocontrol Technologies GmbH & Co. KG, Hundsangen, Germany).

## 3. Results and Discussion

### 3.1. Synthesis

Compounds **1**–**3** were obtained applying the building block approach, using the layering technique [38]. The partial decomposition of the [Cr(C_2_O_4_)_3_]^3−^ anion enabled the release of the oxalate ligand from the coordination sphere of chromium(III), which was consequently coordinated to manganese(II) ions in the reaction mixture during the crystallization process, forming a 1D homometallic oxalate-bridged coordination polymer {[Mn(bpy)(C_2_O_4_)]·1.5H_2_O}*_n_* (**1**). This appearance in which the building block serves as a suitable, additional oxalate source has been recently observed for the tris(oxalato)metalate(III) precursors [26,27], bis(oxalato)chromate(III) [5,39] and oxotris(oxalato)niobate(V) [40,41].

The oxalate release of [Cr(C_2_O_4_)_3_]^3−^ allowed also preparation of heterodimetallic oxalate-containing 1D coordination polymer {[CrCu_3_(bpy)_3_(CH_3_OH)(H_2_O)(C_2_O_4_)_4_][Cu(bpy)Cr(C_2_O_4_)_3_]·CH_2_Cl_2_·CH_3_OH·H_2_O}*_n_* (**2**). It appears that slow diffusion, the solvent mixture used, and the mild reaction conditions utilized herein ensure that this versatile building block surprisingly is an origin of oxalate ligand for the formation of dinuclear [Cu_2_(bpy)_2_(CH_3_OH)(H_2_O)(C_2_O_4_)]^2+^ species of compound **2** further connected. During the crystallization process of heterotrimetallic polymer {[CaCr_2_Cu_2_(phen)_4_(C_2_O_4_)_6_]·4CH_3_CN·2H_2_O}*_n_* (**3**), [Cr(C_2_O_4_)_3_]^3−^ successfully fulfilled its task as a building block—each oxalate group acts as a bridge toward a metal ion, thus forming 3D extended system. 

### 3.2. Infrared (IR) Spectroscopic Study of Compounds ***1***–***3***

The IR spectra of the investigated polymers show the absorption bands that can be attributed to the vibration of the oxalate groups, besides those originating from coordinated *N*-donor ligand (bpy or phen) [42]. The bands of medium intensity found in the region 3600–3000 cm^−1^ originates from the O–H stretching vibrations [*ν*(OH)] of water molecules or those of methanol. Compounds **1**–**3** show characteristic absorption bands of the bis(bidentate), bridging oxalate groups, while compound **2** also shows those of bidentate oxalate group. The absorption bands agreeable to the stretching vibrations of the oxalate groups are summarized in Table 2. Absorption band at 2252 cm^−1^ in the spectra of **3** could be assigned as *ν*(CN) from acetonitrile [42]. The list of all absorption bands of compounds **1**–**3** can be seen in the experimental section.

### 3.3. Molecular and Crystal Structures of Compounds ***1***–***3***

**Figure 1:**
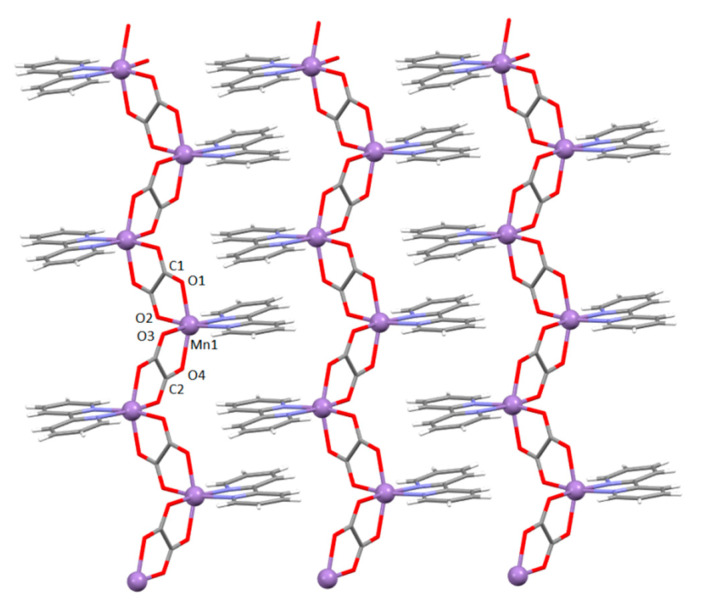
The 1D *zigzag* [Mn(bpy)(C_2_O_4_)]*_n_* chains of polymer **1** in the direction [110]. The aromatic systems of the neighbouring chains are stacked by π-interactions parallel to the plane (001).

The structure of compound {[Mn(bpy)(C_2_O_4_)]·1.5H_2_O}*_n_* (**1**), crystalizing in a monoclinic space group *C*2/*c*, consists of neutral [Mn(bpy)(C_2_O_4_)] units with the manganese(II) ions linked by oxalate groups to form *zigzag* chains extending in the direction [110] (Figure 1), and crystallization water molecules. The asymmetric unit contains, beside manganese(II) atom and bpy ligand, halves of two oxalate bridges (Figure 1 and Figure S1) and one and a half of the crystallization water molecules; one water molecule is located on a twofold axis (therefore, p.p. 0.5), while the other one is disordered about another twofold axis (two positions with p.p. 0.5).

The manganese(II) atom displays distorted octahedral coordination, involving two N atoms from the bipyridine molecule [2.2435(17) and 2.2435(17) Å] and four O atoms from two bridging bis(bidentate) oxalate groups (on average Mn–O = 2.17595 Å). The values of the Mn–N and Mn–O bond lengths (Table S1) are in good agreement with those of similar 1D coordination polymer of manganese(II) ions, crystallizing without water, in space group *Pna*2_1_ [43,44].

The Mn1⋯Mn1*^i^* and Mn1⋯Mn1*^ii^* [symmetry operators: (*i*) −*x*, −*y*, −*z*; (*ii*) 1/2 − *x*, 1/2 − *y*, −*z*] distances across the bridging oxalate group are 5.6249(6) and 5.6656(6) Å, respectively. The shortest distances between two manganese(II) ions from two neighbouring chains is 7.6054(7) Å. 

Water molecule O5 connects two neighbouring chains by hydrogen bonding to oxalate groups, while accepting two C–H∙∙∙O hydrogen bonds from bpy ligands of another two chains (Table S2). Through these interactions, the 1D oxalate-bridged chains are self-assembled into a 3D supramolecular structure.

Bipyridine moieties of neighbouring chains stack in a zipper-like fashion, forming layers parallel to the plane (001) (Figure 1; Table S3). 

**Figure 2:**
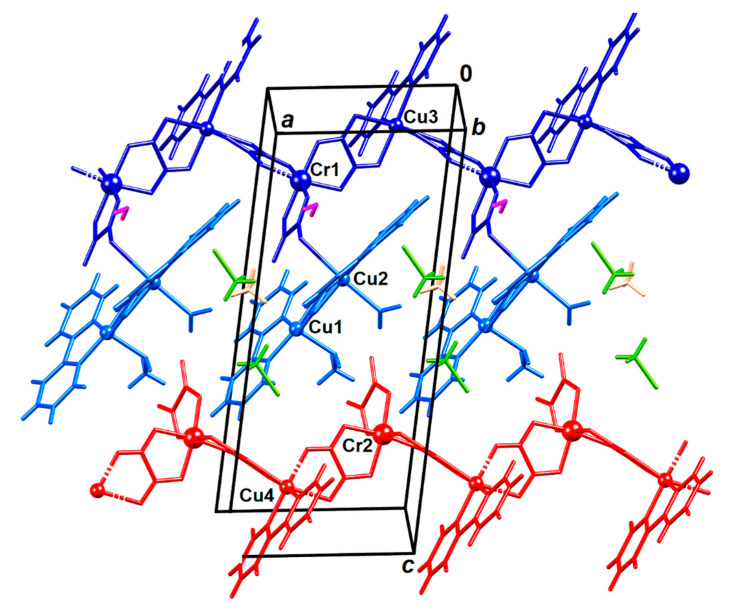
Crystal structure of compound **2**. Symmetry-inequivalent molecules are colour-coded: chain **Cr1–Cu3** is dark blue and its pendant dimeric unit **Cu1–Cu2** is light blue, the **Cr2–Cu4** chain is red, the uncoordinated water molecule is magenta, methanol is green and acetonitrile is orange.

Compound {[CrCu_3_(bpy)_3_(CH_3_OH)(H_2_O)(C_2_O_4_)_4_][Cu(bpy)Cr(C_2_O_4_)_3_]·CH_2_Cl_2_·CH_3_OH·H_2_O}*_n_* (**2**), crystalizing in $P\bar{1}$ space group, comprises a 1D coordination anion [Cu(bpy)Cr(C_2_O_4_)_3_]*_n_^n^*^−^ (**Cr2–Cu4**) with alternating [Cr(C_2_O_4_)_3_]^3^*^−^* and [Cu(bpy)]^2+^ units mutually bridged through an oxalate group, extending in the direction [100] (Figure 2 and Figure S2). Another chain (**Cr1–Cu3**) is similar, but involves homodinuclear unit [Cu(bpy)(CH_3_OH)(*µ*-C_2_O_4_)Cu(bpy)(H_2_O)]^2+^ (**Cu1–Cu2**) coordinated as a pendant group to a terminal oxalate oxygen (Figure 2 and Figure S2). In addition, the asymmetric unit contains one uncoordinated molecule of dichloromethane, methanol and water. 

The copper(II) and chromium(III) atoms in the anionic chains [Cu(bpy)(*µ*-C_2_O_4_)Cr(C_2_O_4_)_2_]*_n_^n−^*(**Cr1–Cu3** and **Cr2–Cu4**) have a usual octahedral coordination: each Cr^3+^ is coordinated by three oxalates, two having bis(bidentate) mode and one bidentate, and atoms Cu^2+^ by four O atoms from two oxalate bridge and two N atoms of 2,2’-bipyridine (Table S4). The Cu1 atom in the dimeric unit [Cu(bpy)(CH_3_OH)(*µ*-C_2_O_4_)Cu(bpy)(H_2_O)]^2+^ (**Cu1–Cu2**) has a distorted square-pyramidal coordination with the bridging oxalate and a bpy moiety forming the basal plane; to the apical positions is bound a methanol molecule. Coordination of Cu2 is a severely Jahn–Teller distorted octahedron with the bridging oxalate and a bpy moiety forming the basal plane; a water molecule and oxalate oxygen atom O16 from the **Cr1–Cu3** unit are in apical positions. The bond Cu2–O16 is very elongated, its length being 2.712(2) Å (Figure 2 and Figure S2); it is significantly longer than the typical Cu–O covalent bond (1.98 Å) [45], but it is shorter than the sum of the van der Waals radii (2.92 Å).

This polymer is the first structurally characterized compound in which copper(II) and chromium(III) centers are connected by bis(bidentate) oxalate group having one-dimensional arrangement [4]; only one heterotrinuclear compound with these metals has been known, prepared by using the [Cr(C_2_O_4_)_3_]^3−^ anion [46]. Two dinuclear [47,48], one trinuclear [49] and one tetranuclear [50] oxalate-bridged compounds containing copper(II) and chromium(III) atoms were found in the literature, but they were synthesized without using tris(oxalato) building block. 

The Cr1···Cu3 and Cr2···Cu4 distances across the oxalate bridges are 5.3236(5) and 5.3552(5) Å, respectively, which are significantly shorter than the corresponding one [5.4605(5) Å] in the known heterotrinuclear compound [46]. The distance between copper(II) ions bridged by oxalate group in **Cu1–Cu2** unit is 5.1504(5) Å. It is somewhat longer than the analogous value (5.086 Å) in the most similar cation found in CSD [4,51]. 

**Figure 3:**
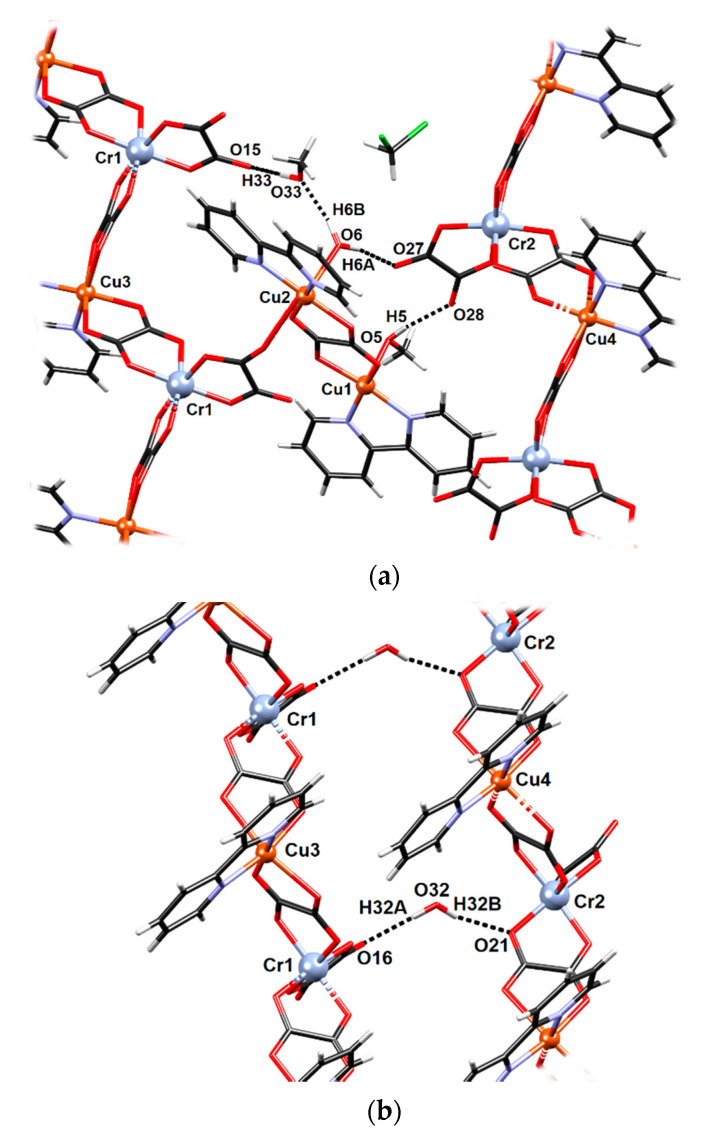
Hydrogen-bonding patterns in **2**: (**a**) dimeric unit **Cu1–Cu2** and an uncoordinated methanol molecule connecting two polymeric chains and (**b**) uncoordinated water molecule connecting the **Cr1–Cu3** and **Cr2–Cu4** chains.

**Figure 4:**
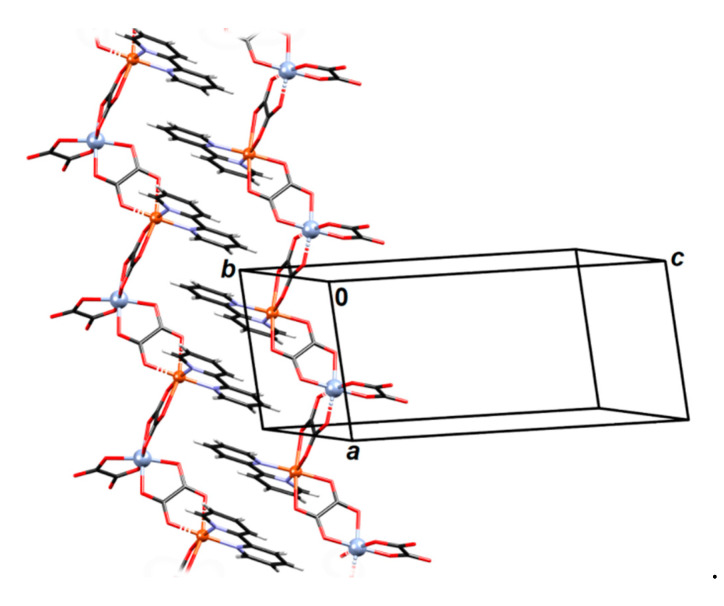
The zipper-like stacking of neighbouring coordination chains in **2**. Chain **Cr1–Cu3** is right and **Cr2–Cu4** is left.

Two polymeric chains are connected through hydrogen bonds: uncoordinated water molecule O32 acts as a proton donor towards two oxalate oxygens (O16 and O21) of two neighbouring symmetry-inequivalent chains (Figure 3; Table S2). A coordinated water molecule acts as a proton donor towards one oxalate oxygen (O27 of the chain **Cr2–Cu4**) and towards the uncoordinated methanol, which in turn acts as a proton donor to O15 of the chain **Cr1–Cu3** (Figure 3). Bipyridine moieties from neighbouring symmetry-inequivalent chains stack in a zipper-like fashion, forming layers parallel to the plane (011). (Figure 4; Table S3).

**Figure 5:**
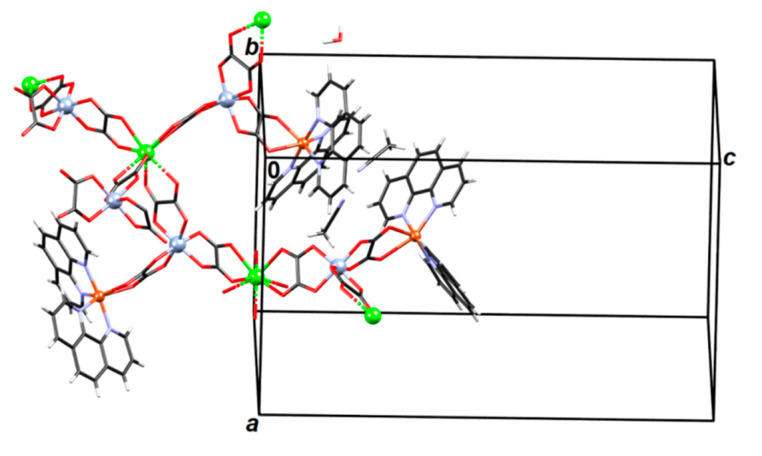
A fragment of a 3D coordination polymer {[CaCr_2_Cu_2_(phen)_4_(C_2_O_4_)_6_]·4CH_3_CN·2H_2_O}*_n_* (**3**).

Compound {[CaCr_2_Cu_2_(phen)_4_(C_2_O_4_)_6_]·4CH_3_CN·2H_2_O}*_n_* (**3**) is a 3D coordination polymer comprising three different metal centers (Ca^2+^, Cr^3+^ and Cu^2+^) oxalate bridged (Figure 5 and Figure S3). The chromium(III) atom is coordinated by six O atoms from three bridging oxalate moieties in octahedral fashion; calcium(II) is located on a twofold axis and is coordinated by eight O atoms from the four oxalates arranged as a dodecahedron, while copper(II) is coordinated by four N atoms of two phenanthroline ligands and one bridging oxalate (Table S5).

**Figure 6:**
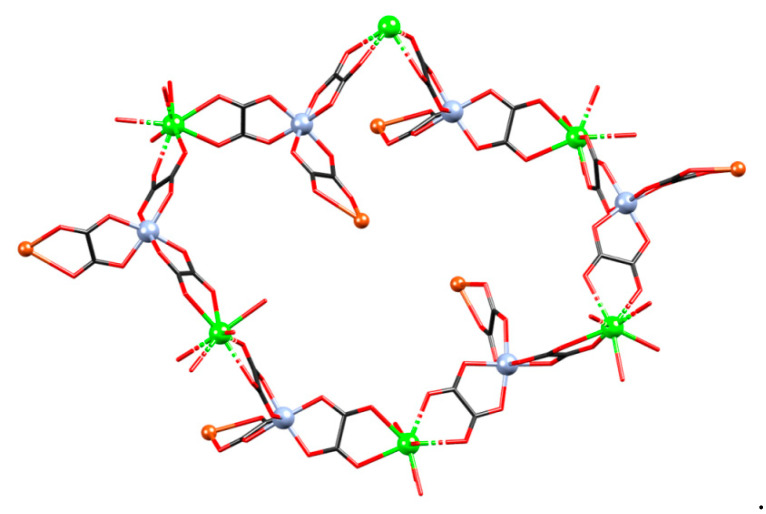
A six-membered ring (part of the **dia**) network in compound {[CaCr_2_Cu_2_(phen)_4_(C_2_O_4_)_6_]·4CH_3_CN·2H_2_O}*_n_* (**3**).

**Figure 7:**
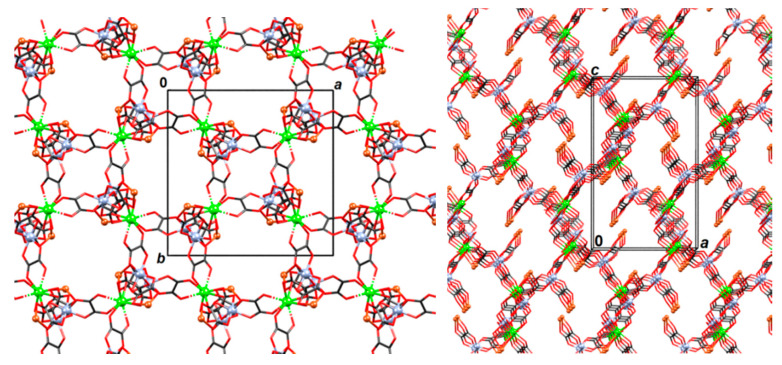
Two views of the 3D coordination polymer {[CaCr_2_Cu_2_(phen)_4_(C_2_O_4_)_6_]·4CH_3_CN·2H_2_O}*_n_* (**3**). Phenanthroline moieties and uncoordinated solvent molecules have been omitted for clarity.

Absolute configurations of Cr^3+^ and Cu^2+^ atoms is *Λ*. Nodes of the 3D network are Ca^2+^ atoms, which are connected with four Cr^3+^ atoms through oxalate bridging ligands (Figure 5); each [Cr(C_2_O_4_)_3_]^3−^ group is bonded to two Ca^2+^ atoms and to one [Cu(phen)_2_]^2+^ unit (Figure 6 and Figure 7). Thus the formed network can be reduced to an underlying graph of diamondoid (**dia**) or (6^6^) topology with Ca^2+^ atoms as nodes and C_2_O_4_–Cr–C_2_O_4_ moieties as links (Figure 5, Figure 6 and Figure S4). In the asymmetric unit one uncoordinated water molecule is present (which acts as a proton donor towards an oxalate oxygen; Table S2) and two acetonitrile molecules. There are also π-interactions between phenanthroline moieties (Table S3).

The distance between Cu^2+^ and Cr^3+^ metal centres bridged by the oxalate ligand is 5.5049(8) Å, while those between Ca^2+^ and Cr^3+^ across the oxalate bridges are 5.7043(7) and 5.7973(9) Å. 

Fascinatingly, a search of the Cambridge Structural Database (CSD) [4] does not find any oxalate-bridged compound containing a combination of these three metals, even non-oxalate-based. 

Simultaneous thermogravimetric analysis (TG) and differential thermal analysis (DTA) have been used for studying the thermal properties of compounds **1**–**3** as crystalline sample in nitrogen atmosphere, up to 1100 °C (Figure S5, Table S6); due to various composition and structural arrangements studied systems show different thermal behavior. All compounds start to decompose almost immediately after the beginning of heating when the evacuation of crystal molecules happened. In all three compounds followed strong exothermic effects can be attributed to the release of *N*-ligands and oxalate groups, when the main loss of mass takes place. The decomposition of **1** ends around 700 °C, when the constant mass is reached, with a black-coloured residue corresponding to Mn_2_O_3_. Probably due to a partial reduction of Cr^6+^ to Cr^3+^, followed by a small mass decrease and a weak endothermic maximum, the degradation of **2** and **3** ends around 960 and 900 °C, respectively, when the mass of the residue matches the mixture of oxides [52].

### 3.4. Magnetization Study of Compounds ***1***–***3***

Temperature dependence of magnetization *M*(*T*) for all three compounds was measured in different magnetic fields, and no splitting between ZFC and FC curves was observed. Field dependence of magnetization *M*(*H*) at different temperatures did not show any hysteresis. Therefore, in both kinds of experiment no irreversibility was observed down to a temperature of 2 K. Moreover, no sharp peaks were observed, excluding thus magnetic long range order in compounds **1**, **2** and **3**.

**Figure 8:**
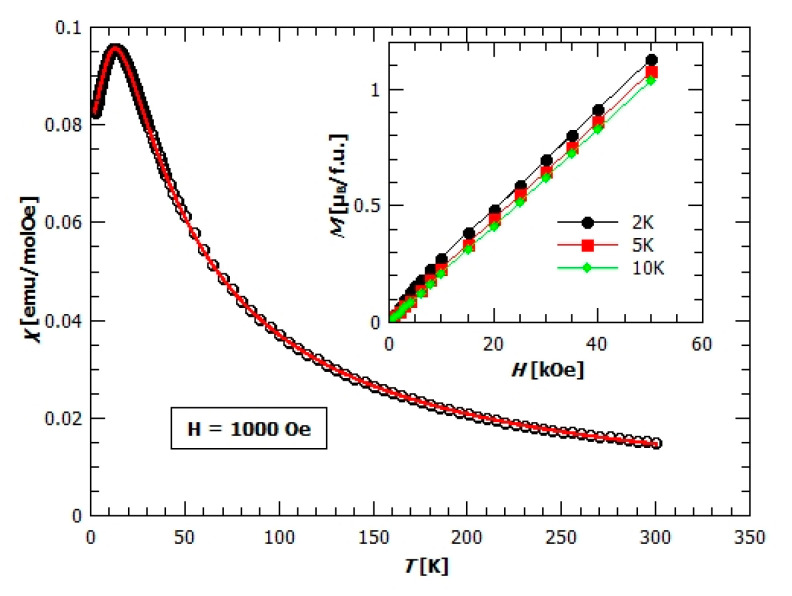
Temperature dependence of the molar magnetic susceptibility *χ* for compound **1** in the field of 1000 Oe. The red solid line represents a 1D model fit as described in the text. Inset: Field dependence of magnetization, *M*(*H*), measured at different temperatures.

Molar magnetic susceptibility corresponding to one manganese ion per formula unit (f.u.) in {[Mn(bpy)(C_2_O_4_)]·1.5H_2_O}*_n_* (**1**) is shown in Figure 8. The inverse susceptibility was fitted first with the Curie–Weiss law:

(1) $$\chi^{-1}\left(T\right)={\left(\chi_{D}+\frac{C}{T-\theta_{CW}}\right)}^{-1}$$

in order to extract preliminary information about the spins and their interactions; χ_D_ is a constant that includes all temperature independent contributions, *C* is the Curie constant, $C=\;\frac{N_{A}\;g^{2}\;\mu_{B}{}^{2}}{3\;k_{B}}\;S\left(S+1\right)$ and $\theta_{CW}=\;\frac{zJ\;S\left(S+1\right)}{3\;k_{B}}$ Weiss temperature that gives the measure of the sum of exchange couplings which determine the local effective field acting on every spin. Other parameters have their usual meaning. Obtained values of the parameters are χ_D_ = 0.00166 ± 0.00009 emu mol^−1^ Oe^−1^, 𝐶 = (4.37 ± 0.03) emu K mol^−1^ Oe^−1^, and *θ_CW_* = (−17.8 ± 0.6) K. The effective magnetic moment is $\mu_{eff}=\sqrt{\frac{3\;k_{B}}{N_{A}\;\mu_{B}{}^{2}}C}=5.915$ corresponding to the magnetic moment of the Mn^2+^ ion in the high spin state (*S* = 5/2 and *g* = 2.00). The negative Weiss temperature suggests antiferromagnetic (AFM) interactions between the magnetic centres and the approximate value of exchange interaction of 𝐽 = −3.05 K, taking into account two nearest neighbours (*z* = 2) bridged by the oxalate ligand along the structural chains as the dominant factor. The existence of AFM interaction could be seen from the *χ*(𝑇) dependence, where relatively broad maximum around 15 K is present, indicating the low dimensional magnetic structure (Figure 8). 

Using the Fisher formula [53] for magnetic chains consisting of large spins (here S = 5/2),

(2) $$\chi_{F}\left(T\right)=\frac{N_{A}g^{2}\mu_{B}{}^{2}S\left(S+1\right)}{3k_{B}T}\;\frac{1+u}{1-u},\;\mathrm{where}\;u=coth\left[\frac{JS\left(S+1\right)}{k_{B}T}\right]-\left[\frac{k_{B}T}{JS\left(S+1\right)}\right]$$

measured data were successfully fitted. The obtained value for the intra-chain super-exchange interaction between the neighbouring Mn^2+^ ions is 𝐽 = (−3.26 ± 0.01) K and 𝑔= (2.050 ± 0.004). It is interesting to note that the value obtained from Curie–Weiss fit is nearly the same as from the Fisher model being a much more accurate description of this system. The Curie–Weiss model is based on figureions along the chain are taken into account, additionally proves the negligible inter-chain magnetic interaction.

Before plotting data (Figure 8) the contribution of paramagnetic impurities and uncompensated Mn^2+^ ions have been subtracted. This amount is *q* = 4.08%, which is determined by fitting the Fisher formula (2) together with the paramagnetic term added:

(3) $$\chi =\left(1-q\right)\;\chi_{F}+q\cdot \frac{N_{A}\mu_{B}{}^{2}}{3k_{B}}\frac{g^{2}S\left(S+1\right)}{T}$$

with S=5/2 as the spin of Mn^2+^ ion, and χ_F_ is from Equation (2).

If we take into account also the inter-chain interactions, within the frame of the mean field approximation,

(4) $$\chi_{z^{\prime }j^{\prime }}=\frac{\chi_{0}}{1-\left(\frac{z^{\prime }j^{\prime }}{N_{A}\mu_{B}{}^{2}}\right)\chi_{0}}$$

where generally χ_0_ is the susceptibility function of non-interacting spin, an insignificant change in parameters and fit-quality with the value of inter-chain interaction of 𝑗′ = (−0.02 ± 0.01) K with z’ = 2 has been obtained. Therefore, the 1D chains containing Mn^2+^ spin can be assumed to be magnetically independent. Magnetic interaction between the chains is negligible because of the large distance of Mn^2+^ ions from neighbouring chains (more than 7Å).

Field dependence of magnetization, *M*(*H*), present in the inset of Figure 8, is typical for the antiferromagnets—slow linear increase of magnetization with the field, far away from saturation. The value of magnetization at 2 K and 5 T is $1.1\;\mu_{B}/f.u.$, which is far lower than the expected value of $5\;\mu_{B}/f.u.$ for the spin 5/2.

The magnetic properties of similar oxalate-bridged 1D coordination polymer of manganese(II) were also studied [43] and interaction was found to be −1.72, K with inter-chain Weiss parameter of −0.04 K. Compared to the known compound, **1** seems as being closer to the ideal 1D Heisenberg antiferromagnetic chain with stronger intra-chain interaction. This is due to a slight difference in the structural packing of these two compounds, as a consequence of the existence of crystalline water in **1**.

Chains containing manganese ions have been of interest for a long time, and they were synthesized using other superexchange-bridges. One example is chloride-bridged chain with dimethylammonium ions separating them [54], having considerably stronger intra-chain interaction of −6.9 K and inter-chain interaction of −0.5 K. Although this *J* indicates a stronger interaction than in **1**, considerably weaker interaction between the chains (−0.02 K) makes our system much closer to the ideal 1D Heisenberg antiferromagnet. 

**Figure 9:**
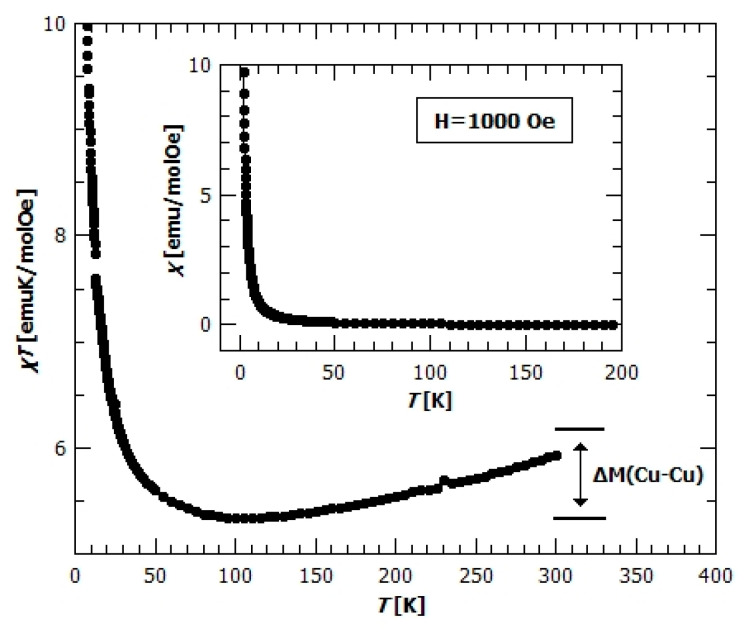
Temperature dependence of χT for the compound **2** in the field of 1000 Oe. Inset: temperature dependence of susceptibility in 1000 Oe.

Temperature dependence of susceptibility, *χ*(*T*), of compound {[CrCu_3_(bpy)_3_(CH_3_OH)(H_2_O)(C_2_O_4_)_4_][Cu(bpy)Cr(C_2_O_4_)_3_]·CH_2_Cl_2_·CH_3_OH·H_2_O}*_n_* (**2**) in field of 1000 Oe (inset in Figure 9) shows no phase transition, but the rapid/sharp increase at temperatures below 30 K indicates the presence of ferromagnetic interactions (FM). From the crystal structure we can assume that it belongs to low-dimensional magnetic structures, i.e., magnetic chains of copper(II) and chromium(III) ions bridged by oxalate ligand. Fitting the reciprocal susceptibility data with Curie-Weiss law, equation (1), following parameters: 𝐶 = (4.43 ± 0.02) emu K mol^−1^ Oe^−1^, $\theta_{CW}=\left(9.3\pm 0.3\right)\;K$, and $\chi_{D}=\left(0.00450\pm 0.00005\right)$ emu mol^−1^ Oe^−1^ have been obtained. One formula unit contains 4 Cu^2+^ and 2 Cr^3+^ ions, so that the Curie constant should be $C=\frac{N_{A}\mu_{B}{}^{2}}{3k_{B}}\underset{i}{\sum }g_{i}{}^{2}\;S_{i}\left(S_{i}+1\right)=5.27$ emu K mol^−1^ Oe^−1^, where *g*-factors $g_{Cu}=2.11$, and $g_{Cr}=1.96$ have been taken [55]. Measured value is somewhat lower, indicating that not all of the spins participate in this sum of free paramagnetic ions’ contributions. This discrepancy between calculated by the Curie constant and the observed value will be explained below, by analysing the temperature-dependence of χT. However, a positive Weiss temperature surely confirms the existence of FM interactions with the average intra-chain interaction of the order between 1 and 10. 

In Figure 9, the temperature dependence of χT shows the minimum at 100 K, where, after the initial rapid decline with increasing temperatures, χT starts to slowly increase. This increase can be explained by looking more closely at the crystal structure of compound **2**; one formula unit contains 4 Cu^2+^ and 2 Cr^3+^ ions, from two chains [Cu(bpy)(*µ-*C_2_O_4)_Cr(C_2_O_4_)_2_]*_n_^n−^*(**Cr1–Cu3** and **Cr2–Cu4**) and one dimeric unit [Cu(bpy)(H_2_O)(*µ*-C_2_O_4_)Cu(bpy)(CH_3_OH)]^2+^ (**Cu1–Cu2**) (Figure 2). This homodinuclear cation can be regarded as magnetically independent from the other magnetic centres, since a terminal oxalate oxygen atom from the **Cr1–Cu3** chain coordinated to copper(II) ion of this dinuclear unit, does not provide the exchange bridging path. Such dimers usually have a strong antiferromagnetic interaction [26,27,56,57], so that the spin of a dimer is 0. Assuming that is the case here, the increase of χT as the temperature rises can be understood as the start of the decoupling of a dimer with very strong antiferromagnetic exchange intradimer interaction (few hundreds of Kelvins). From this viewpoint, the underestimated Curie constant obtained from the Curie–Weiss fit is understandable. By measuring the higher temperatures, where the dimer (**Cu1**–**Cu2**) is decoupled, the calculated value of Curie constant would be obtained, but then the compound would not be thermally stable. In Figure 9, the contribution of the decoupling dimer is indicated with the ΔM(Cu–Cu), and it can be seen that at 300 K the χT does not yet achieve the value where all the spins in the formula unit are decoupled (2 Cr^3+^ and 4 Cu^2+^ ions per f.u.), but as it still rises it can be assumed that at higher temperatures it will saturate at this value.

**Figure 10:**
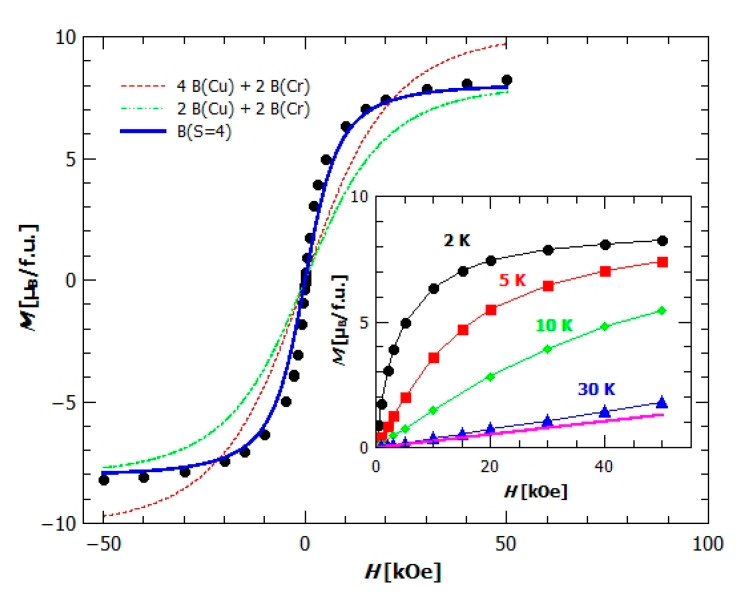
Field dependence of magnetization of compound **2**, with plotted Brillouin functions B for different possible situations (explained in text). Inset: field dependence of magnetization at different temperatures, with the pink line representing the B function for independent spins in the formula unit.

Field dependence of magnetization, *M*(*H*), shows rapid increase of magnetization at lower fields and saturation at high fields (Figure 10). From the value of saturation magnetization, $\mu_{s}=8.2\;\mu_{B}/f.u.$, it could be concluded that the ground state spin projection in one formula unit is 4. This shows that all spins in both chains (**Cr1–Cu3** and **Cr2–Cu4**) point in the same direction (direction of applied magnetic field) in their ground state and confirms the persistence of strong AFM dimers (**Cu1–Cu2**). To check this assumption we have plotted (Figure 10) the Brillouin function for different cases, first for the case in which all magnetic ions are magnetically independent (red dashed line), then the case where the spin in dimer is 0 and two Cr^3+^ and two Cu^2+^ ions are independent (green dash-dot line) and, finally, the case where we have one spin of 4 in formula unit (Brillouin function for spin S = 4) (blue line). The best agreement with the data shows the Brillouin function for spin 4, but the measured susceptibility is still above the Brillouin function, indicating strong enough FM interactions to produce large spin correlations along [Cu(bpy)(*µ-*C_2_O_4)_Cr(C_2_O_4_)_2_]*_n_^n−^*chains. In the inset of Figure 10 the field dependence of magnetization *M*(*H*) is shown for temperatures 2, 5, 10 and 30 K and, as expected, the field dependence is becoming linear with increasing temperature, but even at temperature of 30 K the measured magnetization is higher than that for a case of paramagnetic spins of two Cr^3+^ and two Cu^2+^ (pink line in the inset of Figure 10 represents the sum of Brillouin functions for Cr^3+^ and Cu^2+^ ions), and this difference comes from the finite *M*(*H*) response of **Cu1–Cu2** dimer. 

**Figure 11:**
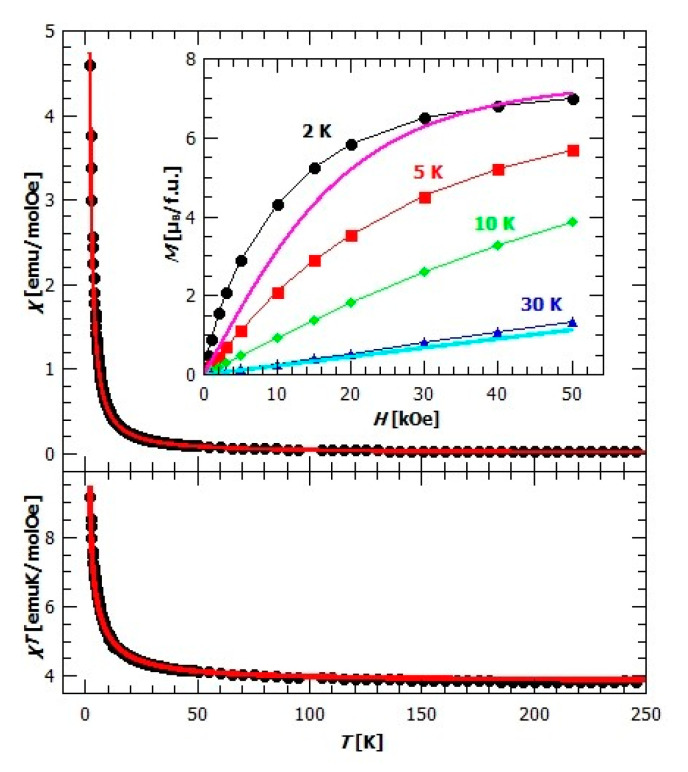
Up: temperature dependence of susceptibility χ for compound **3** in field of 1000 Oe. The red line represents fitted curve. Inset: field dependence of magnetization at different temperatures. Pink and blue lines represent the sum of Brillouin functions for spins of Cu^2+^ and Cr^3+^. Down: temperature dependence of χT in the field of 1000 Oe. The red line is fitting curve.

From the crystal structure of compound {[CaCr_2_Cu_2_(phen)_4_(C_2_O_4_)_6_]·4CH_3_CN·2H_2_O}*_n_* (**3**) (Figure 5), it can be assumed the existence of magnetic dimers of Cu^2+^ and Cr^3+^ ions connected through the oxalate bridge, since Ca^2+^ is diamagnetic, thereby stopping the propagation of further exchange pathways. Temperature dependence of susceptibility shows no phase transition and χT(T) suggests the ferromagnetic interaction between magnetic moments (Figure 11). Magnetic measurements were modelled using PHI software (Chilton Group, Manchester, UK.) [58] with Hamiltonian for a [Cu^II^Cr^III^] dimer:

(5) $$H=-JS_{Cu}S_{Cr}+\mu_{B}\left(g_{Cu}S_{Cu}+g_{Cr}S_{Cr}\right)\cdot B+D_{Cr}\left(S_{Cr}{{}_{z}}^{2}-\frac{1}{3}S_{Cr}\left(S_{Cr}+1\right)\right)$$

where the zero-field splitting (ZFS) of Cr^3+^ was also taken into account with component characteristic for axially distorted octahedral coordination. It has been found that the exchange constant is J=(7.1±0.1) K, with *g*-factors $g_{Cu}=\left(2.0000\pm 0.0005\right)$, $g_{Cr}=\left(1.815\pm 0.002\right)$, and the axial ZFS parameter $D_{Cr}=\left(-2.8\pm 0.1\right)\;K$. An additional check of this fit was performed with a program developed in Python, which was as successful as in other complex cases [59]. Ferromagnetic super exchange in Cu^II^–C_2_O_4_–Cr^III^ magnetic units can be understood within the orthogonality of the magnetic orbitals. Namely, Cu^2+^ ion has an axially distorted configuration, and one unpaired electron on the $d_{x^{2}-y^{2}}$ orbital oriented toward the 4 nearest atoms (N1, N2, O2 and N4) in the coordination environment (Figure S3). It interacts with the occupied orbitals of the oxalate group producing a magnetic orbital of *σ*-character. Ion Cr^3+^ in the octahedral configuration has three unpaired electrons on the $d_{xy}$, $d_{xz}$ and $d_{yz}$ orbitals interacting with other occupied orbitals of the oxalate group, producing magnetic orbitals of π-character. The overall superexchange interaction is expected to be ferromagnetic due to orthogonality of all these magnetic orbitals [48]. In other compounds with oxalate-bridged [Cu^II^Cr^III^] dimer where the symmetry is such that the ferromagnetic coupling can be achieved due to orthogonality of magnetic orbitals, the ferromagnetic exchange is of comparable value [46,47,48].

Using the mean field approach within PHI software for interactions between the dimers (Equation (4)), it was found that the small intermolecular interaction between dimers does exist, in amount zj=(0.078±0.001) K. This could mean that despite the large distance between dimers and the presence of diamagnetic Ca^2+^, dimers still interact and interaction could come through the small but finite spin polarization of Ca^2+^ orbitals which could produce super-exchange over this nearly diamagnetic bridge, as was already observed and also studied theoretically in the –Cr^III^–O–Nb^V^–O–Cr^III^– complex [60,61]. This motivates further investigation and the calculation of the spin densities, which is beyond the scope of the present work.

The existence of ferromagnetic interaction could also be seen from the field dependence of magnetization, inset in Figure 11, as the measured magnetization at 2K (black dots), is above the values expected for the independent spins and given by the sum of Brillouin functions for spin 1/2 and 3/2 (pink line). As expected, with the increasing temperature, the difference between the measured magnetization and the magnetization of the independent spins decreases, but is still present at 30 K, meaning that dimers are not completely decoupled yet.

### 3.5. Electrical Study of Compounds ***1***–***3***

**Figure 12:**
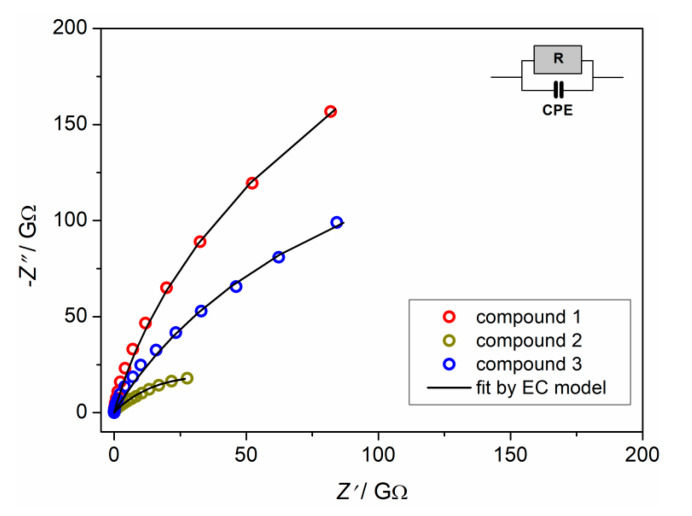
Complex impedance plot and corresponding equivalent circuit (EC) for compounds **1**–**3** at room temperature.

The complex impedance plots of compounds **1**–**3** at RT are shown in Figure 12. Compounds **1** and **3** exhibit arcs at higher values of impedance indicating low electrical conductivity. On the other hand, compound **2** shows a better defined impedance semicircle at lower values of impedance. Obtained impedance data can be approximated well by the equivalent electrical circuit consisting of a parallel combination of resistor and constant phase element (CPE). The fitting parameters for all compounds are given in Table S7. The values of the electrical resistance (*R*) obtained from the fitting procedure and electrode dimensions (*d* is sample thickness and *A* is electrode area) were used to calculate the DC conductivity for all compounds according to the relation *σ*_DC_ = *d*/(*R*×*A*).

Compounds **1** and **3** exhibit similar behavior with low values of DC conductivity [≈2.2 × 10^−12^ (Ω cm)^−1^] at room temperature. On the other hand, compound **2** shows higher electrical conductivity, reaching the value of 1.33 × 10^−11^ (Ω cm)^−1^ at RT (Table S7).

Compound **2** has complex anion and complex cation which is not the case for compounds **1** and **3**. It seems that the molecular environment in compound **2** enhances the ease of proton transfer and further formation of continuous conduction pathways for the uninterrupted electrical transport. Furthermore, coordination solvents in compound **2**, water and methanol, enable hydrogen bonds to network which, probably, additionally increases DC conductivity, in comparison to complexes **1** and **3**. Moreover, complexes **1** and **3,** having a lack of hydrogen bonds (see Section 3.3 and Table S2), show similar DC conductivity.

## 4. Conclusions

In summary, three novel oxalate-bridged coordination polymers were obtained using [Cr(C_2_O_4_)_3_]^3−^ as building block: two 1D (**1** and **2**) and one 3D (**3**), of which one is homo- ([Mn^II^]; **1**), one heterodi- ([Cu^II^Cr^III^]; **2**) and one heterotrimetallic ([Ca^II^Cu^II^Cr^III^]; **3**), and their magnetic and electrical properties were investigated. Due to the partial decomposition of the precursor of chromium(III) used during the crystallization process, compounds **1** and **2** were formed. In **3**, each oxalate group of the precursor acts as a bridge toward the metal ion, thus forming 3D extended system.

Magnetic properties of investigated compounds showed very different behaviors. In compound **1**, the antiferromagnetic Mn-chains with 𝐽 = (−3.26 ± 0.01) K and almost no inter-chain interaction, 𝑗′ = (−0.02 ± 0.01) K, are closer to the ideal 1D Heisenberg antiferromagnetic chain than the other known chains containing manganese(II). The magnetism of compound **2** showed the existence of strongly coupled antiferromagnetic copper dimers (Cu−C_2_O_4_−Cu), independent from the rest of the magnetic ions which couple ferromagnetically within the Cu−C_2_O_4_−Cr chains. Compound **3** showed dimeric behavior, with Cu^2+^ and Cr^3+^ ions coupled ferromagnetically [J=(7.1±0.1) K], but with small inter-dimer interactions (zj=(0.078±0.001) K) which could come from the small spin polarization of Ca^2+^ orbitals producing thereby a super-exchange over this nearly diamagnetic bridge.

## Figures and Tables

**Table 1:** Crystallographic data and structure refinement details for compounds **1**–**3**.

Compound	1	2	3
Empirical formula	C_12_H_9_MnN_2_O_5_	C_57_H_46_Cl_2_Cr_2_Cu_4_N_8_O_32_	C_68_H_48_CaCr_2_Cu_2_N_12_O_26_
Formula wt./g mol^−1^	324.15	1784.12	1720.36
Colour	yellow	green	dark green
Crystal dimensions/mm	0.23 × 0.14 × 0.09	0.80 × 0.50 × 0.20	0.25 × 0.15 × 0.05
Space group	*C2/c*	*P* $\bar{1}$	*P*4_1_2_1_2
*a*/Å	14.3981(3)	8.7537(2)	16.1956(1)
*b*/Å	12.7019(2)	18.5551(6)	16.1956(1)
*c*/Å	16.5031(5)	21.4099(7)	26.5448(2)
*α*/°	90	72.907(3)	90
*β*/°	118.536(3)	81.372(2)	90
*γ*/°	90	85.676(2)	90
*Z*	8	2	4
*V*/Å^3^	2651.49(13)	3284.50(18)	6962.63(10)
*D*_calc_/g cm^−3^	1.624	1.802	1.641
*μ*/mm^−1^	8.358	5.677	4.651
F(000)	1312	1796	437
*Θ* range/°	6.42–75.85	3.74–75.98	3.19–75.28
*T*/K	293(2)	100(2)	293(2)
Range of *h*, *k*, *l*	−18 < h < 16;	−10 < h < 10	−20 < h < 13
	−15 < k < 15;	−23 < k < 23	−20 < k < 19
	−16 < l < 20	−26 < l < 26	−26 < l < 33
Reflections collected	6468	34327	22852
Independent reflections	2705	13553	7164
Observed reflections (*I* ≥ 2*σ*)	2448	12642	6857
*R*_int_	0.0230	0.0326	0.0377
*R*, w*R* [*I* ≥ 2*σ*]	0.0321	0.0390	0.0351
w*R* [*I* ≥ 2*σ*]	0.0876	0.1057	0.0937
Goodness-of-fit	1.033	1.048	1.042
H atom treatment	Mixed	Mixed	Mixed
No. of parameters, restraints	200, 2	974, 21	505, 6
Δ*ρ*_max_, Δ*ρ*_min_, Δ*ρ*_rms_ (eÅ^−3^)	0.201; −0.276; 0.045	1.249; −1,574; 0.082	0.299; −0.432; 0.052

**Table 2:** Selected absorption bands (cm^−1^) of the oxalate groups in the infrared spectra of compounds **1**–**3.**

Compound	Bidentate Oxalate Group			Bis(bidentate) Oxalate Group		
	*ν_a_*_s_(CO)	*ν*_s_(CO)	*δ*(OCO)	*ν*_as_(CO)	*ν*_s_(CO)	*δ*(OCO)
**1**	–	–	–	1673	1310	793
**2**	1702, 1679, 1634	1389, 1252	814	1664	1293	773
**3**	–	–	–	1664	1282	781

## Supplementary Materials

The following are available online at https://www.mdpi.com/1996-1944/13/23/5341/s1, Table S1: Bond lengths (Å) and angles (°) for the metal coordination sphere in coordination polymer **1**, Table S2: Geometric parameters of hydrogen bonds (Å, °) in coordination polymers **1**, **2** and **3**, Table S3: Geometric parameters of π-stacking (Å, °) in coordination polymers **1**, **2** and **3**, Table S4: Bond lengths (Å) and angles (°) for the metal coordination spheres in coordination polymer **2**, Table S5: Bond lengths (Å) and angles (°) for the metal coordination spheres in coordination polymer **3**. Symmetry operators: (*i*) 1/2 + *x*, 3/2 − *y*, −1/4 − *z*; (*ii*) −1/2 − *y*, ½ − *x*, −1/4 + *z*; (*iii*) 1 − *y*, 1 − *x*, −1/2 − *z*, Table S6: Thermoanalytical data for compounds **1**–**3**, Table S7: The best fitting parameters obtained from equivalent circuit modeling of complex impedance spectra measured at room temperature for compounds **1**–**3**, and calculated DC conductivity, Figure S1: ORTEP-3 diagram of compound {[Mn(bpy)(C_2_O_4_)]·1.5H_2_O}*_n_* (**1**) with atom numbering scheme (only asymmetric unit is numbered). Displacement ellipsoids are drawn for the probability of 50% and hydrogen atoms are shown as spheres of arbitrary radii, Figure S2: ORTEP-3 diagram of (a) dimeric unit **Cu1–Cu2**, (b) 1D chain **Cr1–Cu3** and (c) 1D chain **Cr2–Cu4** in compound **2** with atom numbering scheme. Displacement ellipsoids are drawn for the probability of 50% and hydrogen atoms are shown as spheres of arbitrary radii, Figure S3: ORTEP-3 diagram of asymmetric unit of compound **3** with atom numbering scheme (uncoordinated water and acetonitrile molecules have been omitted for clarity). Displacement ellipsoids are drawn for the probability of 50% and hydrogen atoms are shown as spheres of arbitrary radii, Figure S4: Underlying graph with **dia** topology in coordination polymer **3**. Nodes represent Ca atoms, Figure S5: The thermogravimetric analysis (TG) and differential thermal analysis (DTA) curves for compounds **1**–**3** measured in nitrogen atmosphere.
