# Supplementary material for: Magnetic and Electrical Behaviors of the Homo- and Heterometallic 1D and 3D Coordination Polymers Based on the Partial Decomposition of the [Cr(C2O4)3]3− Building Block
Source: Materials (Basel). 2020 Nov 25;13(23):5341. doi: 10.3390/ma13235341 (PMC7728352; doi:10.3390/ma13235341)
Supplement: Supplementary file 1 [file materials-13-05341-s001.pdf]

materials

Supplementary Materials

Magnetic and Electrical Behaviors of the Homo- and Heterometallic 1D and 3D Coordination Polymers Based on the Partial Decomposition of the $[Cr(C_2O_4)_3]^{3-}$ Building Block

Lidija Kanižaj [1], Pavla Šenjug [2], Damir Pajić [2], Luka Pavić [1], Krešimir Molčanov [1] and Marijana Jurić [1,*]

[1] Ruđer Bošković Institute, Bijenička cesta 54, 10000 Zagreb, Croatia; Lidija.Kanizaj@irb.hr (L.K.); lpavic@irb.hr (L.P.); Kresimir.Molcanov@irb.hr (K.M.)
[2] Department of Physics, Faculty of Science, University of Zagreb, Bijenička cesta 32, 10000 Zagreb, Croatia; psenjug@phy.hr (P.S); dpajic@phy.hr (D.P.)
* Correspondence: Marijana.Juric@irb.hr; Tel.: +385 1 456 1189

Received: 19 October 2020; Accepted: 19 November 2020; Published: 25 November 2020

Table S1. Bond lengths (Å) and angles (°) for the metal coordination sphere in coordination polymer **1**.

Mn1–O1	2.1692(14) 2.1689(14)	O1–Mn1–O12	76.58(5)
Mn1–O2	2.1941(15)	O1–Mn1–O4	160.70(6)
Mn1–O3	2.1716(14)	O2–Mn1–O4	91.29(6)
Mn1–O4	2.2435(17) 2.2325(16)	O2–Mn1–O3	98.26(7)
Mn1–N1		O1–Mn1–O3	90.81(6)
Mn1–N2		O4–Mn1–O3	75.93(5)
		O2–Mn1–N2	165.66(6)
		O1–Mn1–N2	96.77(6)
		O4–Mn1–N2	98.18(6)
		O3–Mn1–N2	94.48(6)
		O2–Mn1–N1	94.09(6)
		O1–Mn1–1	92.70(6)
		O4–Mn1–N1	103.23(6)
		O3–Mn1–N1	167.63(6)
		N2–Mn1–N1	73.32(6)

Table S2. Geometric parameters of hydrogen bonds (Å, °) in coordination polymers **1, 2** and **3.**

	D–H / Å	H···*A* / Å	*D*···*A* / Å	*D*–H···*A* / º	Symm. op. on *A*
**1**					
O5–H5···O4	0.98(3)	2.03(4)	2.941(2)	153(3)	*x, y, z*
C11–H11···O5	0.93	2.54	3.357(3)	147	1−*x*, −*y*, −*z*
**2**					
O5–H5···O28	0.82(3)	1.90(3)	2.682(3)	161(3)	*x, y, z*
O6–H6A···O27	0.92(3)	1.86(3)	2.774(3)	168(3)	*x, y,z*
O6–H6B···O33	0.93(3)	1.83(3)	2.733(3)	165(4)	*x, y, z*
O32–H32A···O16	0.85	2.02	2.874(3)	178	−*x*, 1−*y*, 1−*z*
O32–H32B···O21	0.85	2.07	2.888(3)	160	−1+*x*, *y*, *z*
O33–H33A···O15	0.94(5)	1.78(5)	2.720(3)	177(4)	−1+*x*, *y*, *z*
**3**					
O13–H13B···O10	0.96(4)	2.25(6)	2.940(6)	127(6)	−1+*y*, 1+*x*, −*z*

Table S3. Geometric parameters of π-stacking (Å, °) in coordination polymers **1**, **2** and **3.**

π···π	Cg[a]···Cg / Å	α[b]	β[c]	Cg···plane(Cg2) / Å	Offset/ Å[d]	Symm. op. on Cg2
**1**						
N1→C7···N2→C12	3.7707(14)	7.89(12)	18.8	3.7030(10)	1.213	1/2–*x*, –1/2–*y*, –*z*
N2→C12···N2→C12	3.9772(15)	0.00(12)	26.6	3.5577(10)	1.778	1–*x*, –*y*, –*z*
**2**						
Cu1→N2···N1→C5	3.6662(14)	0.31(11)	20.6	3.4246(9)	1.293	2–*x*, 2–*y*, 1–*z*
Cu3→N6···N7→C55	3.7858(14)	6.36(11)	24.1	3.5889(9)	1.548	1+*x*, 1+*y*, –1+*z*
N1→C5···N1→C5	3.6178(15)	0.00(12)	18.4	3.4322(10)	1.144	2–*x*, 2–*y*, 1–*z*
N4→C20···N4→C20	3.9934(15)	0.00(13)	36.6	3.2071(11)	2.379	1–*x*, 1–*y*, 1–*z*
N5→C39···Cu4→N8	3.9794(14)	4.31(11)	22.7	3.5892(11)	1.538	1+*x*, 1+*y*, –1+*z*
N5→C39···N8→C50	3.9900(15)	2.15(12)	24.1	3.6317(11)	1.629	1+*x*, 1+*y*, –1+*z*
N6→C34···N7→C55	3.7744(15)	5.59(12)	21.8	3.3526(10)	1.403	1+*x*, 1+*y*, –1+*z*
N6→C34···N8→C50	3.9907(15)	2.81(12)	29.8	3.5549(10)	1.984	*x*, 1+*y*, –1+*z*
**3**						
N3→C23···C10→C17	3.739(2)	6.7(2)	16.3	3.6686(18)	1.050	1/2–x, 1/2+y, 1/4–z
C10→C17···C10→C17	3.782(2)	17.72(18)	11.4	3.7072(16)	0.748	1–x, 1–y, 1/2–z
C10→C17···C22→C29	3.880(2)	8.9(2)	30.4	3.6055(16)	1.962	1/2–x, –1/2+y, 1/4–z

[a] Cg = centre of gravity of the aromatic ring.

[b] α = angle between planes of two interacting rings.

[c] β = angle between Cg···Cg line and normal to the plane of the first interacting ring.

[d] Offset can be calculated only for the strictly parallel rings (α = 0.00°). For slightly inclined rings ($\alpha \leq 5°$) an approximate value is given.

Table S4. Bond lengths (Å) and angles (°) for the metal coordination spheres in coordination polymer **2**.

Cr1-O14	1.9488(18)	Cr2-O26	1.9530(18)	Cu3-N6	1.990(2)
Cr1-O13	1.9555(19)	Cr2-O25	1.9598(17)	Cu3-N5	1.997(2)
Cr1-O17	1.9854(18)	Cr2-O21	1.9744(19)	Cu3-O9	2.0057(19)
Cr1-O12	1.9854(17)	Cr2-O24	1.9805(18)	Cu3-O8	2.0287(18)
Cr1-O18	1.9949(17)	Cr2-O23	1.9894(17)	Cu3-O10	2.2791(18)
Cr1-O11	1.9951(18)	Cr2-O22	1.9961(17)	Cu3-O7	2.3329(18)
O14-Cr1-O13	83.74(8)	O26-Cr2-O25	82.57(7)	N6-Cu3-N5	81.87(9)
O14-Cr1-O17	173.19(8)	O26-Cr2-O21	177.31(8)	N6-Cu3-O9	175.46(9)
O14-Cr1-O12	96.26(8)	O26-Cr2-O24	91.35(8)	N6-Cu3-O8	97.24(8)
O14-Cr1-O18	93.13(7)	O26-Cr2-O23	91.13(7)	N6-Cu3-O10	101.33(7)
O14-Cr1-O11	94.37(8)	O26-Cr2-O22	96.07(7)	N6-Cu3-O7	85.13(7)
O13-Cr1-O17	90.80(8)	O25-Cr2-O21	95.36(8)	N5-Cu3-O9	93.59(8)
O13-Cr1-O12	178.49(8)	O25-Cr2-O24	90.58(7)	N5-Cu3-O8	169.20(8)
O13-Cr1-O18	92.38(7)	O25-Cr2-O23	170.92(8)	N5-Cu3-O10	103.90(7)
O13-Cr1-O11	95.91(7)	O25-Cr2-O22	99.24(7)	N5-Cu3-O7	91.31(7)
O17-Cr1-O12	89.31(8)	O21-Cr2-O24	90.39(8)	O9-Cu3-O8	87.25(8)
O17-Cr1-O18	83.02(7)	O21-Cr2-O23	91.13(8)	O9-Cu3-O10	79.55(7)
O17-Cr1-O11	90.22(8)	O21-Cr2-O22	82.54(7)	O9-Cu3-O7	95.18(7)
O12-Cr1-O18	89.14(7)	O24-Cr2-O23	83.01(7)	O8-Cu3-O10	86.84(7)
O12-Cr1-O11	82.58(7)	O24-Cr2-O22	168.36(7)	O8-Cu3-O7	77.90(7)
O18-Cr1-O11	169.38(7)	O23-Cr2-O22	87.88(7)	O10-Cu3-O7	164.12(7)
Cu1-N1	1.974(2)	Cu2-N3	1.979(2)	Cu4-N8	1.984(2)
Cu1-O1	1.9749(17)	Cu2-O4	1.9807(18)	Cu4-O31	1.9912(17)
Cu1-N2	1.977(2)	Cu2-O3	1.9873(18)	Cu4-N7	1.995(2)
Cu1-O5	2.2086(18)	Cu2-O6	2.2841(18)	Cu4-O20	2.0171(18)
O2-Cu1-N1	159.71(8)	N4-Cu1-N3	82.52(9)	Cu4-O19	2.3275(18)
O2-Cu1-O1	84.96(7)	N4-Cu1-O4	95.96(8)	Cu4-O30	2.3405(19)
O2-Cu1-N2	93.77(8)	N4-Cu1-O3	169.31(8)	N8-Cu4-O31	177.50(8)
O2-Cu1-O5	99.94(7)	N4-Cu1-O6	93.75(8)	N8-Cu4-N7	81.80(9)
N1-Cu1-O1	96.72(8)	N3-Cu1-O4	178.18(8)	N8-Cu4-O20	94.02(8)
N1-Cu1-N2	81.96(8)	N3-Cu1-O3	96.74(8)	N8-Cu4-O19	96.70(7)
N1-Cu1-O5	100.23(8)	N3-Cu1-O6	89.34(7)	N8-Cu4-O30	100.19(7)
O1-Cu1-N2	172.60(8)	O4-Cu1-O3	84.94(7)	O31-Cu4-N7	96.28(8)
O1-Cu1-O5	91.40(7)	O4-Cu1-O6	89.77(7)	O31-Cu4-O20	88.08(8)
N2-Cu1-O5	96.01(7)	O3-Cu1-O6	96.91(7)	O31-Cu4-O19	85.05(7)
				O31-Cu4-O30	78.42(7)
				N7-Cu4-O20	172.13(8)
				N7-Cu4-O19	95.24(7)
				N7-Cu4-O30	97.17(7)
				O20-Cu4-O19	78.57(7)

		O20-Cu4-O30	90.11(7)
		O19-Cu4-O30	160.28(7)

Table S5. Bond lengths (Å) and angles (°) for the metal coordination spheres in coordination polymer **3**. Symmetry operators: (*i*) 1/2 + *x*, 3/2 – *y*, –1/4 – *z*; (*ii*) –1/2 – *y*, ½ – *x*, –1/4 + *z*; (*iii*) 1–*y*, 1 – *x*, –1/2 – *z*.

Cu1–N1	2.006(3)	Cr1–O1	1.984(3)	Ca1–O6	2.487(3)
Cu1–N2	2.092(3)	Cr1–O3	1.980(3)	Ca1–O6^{iii}	2.487(3)
Cu1–N3	2.144(3)	Cr1–O5	1.992(3)	Ca1-O8	2.441(3)
Cu1–N4	2.001(3)	Cr1–O7	1.969(3)	Ca1–O8^{iii}	2.441(3)
Cu1–O2	2.178(3)	Cr1–O9	1.990(3)	Ca1–O10^{i}	2.603(3)
Cu1–O4	2.484(3)	Cr1–O11	1.964(3)	Ca1–O10^{ii}	2.603(3)
				Ca1–O12^{i}	2.406(3)
N1–Cu1–N2	81.12(13)	O11-Cr1-O7	91.40(11)	Ca1–O12^{ii}	2.406(3)
N1–Cu1–N3	93.79(13)	O11-Cr1-O3	87.97(11)		
N1–Cu1–O2	86.52(12)	O11-Cr1-O1	91.86(11)	O12^{i}-Ca1-O12^{ii}	121.67(11)
N4–Cu1–N2	99.33(13)	O11-Cr1-O9	82.98(11)	O12^{i}-Ca1-O8^{iii}	69.40(10)
N4–Cu1–N1	174.05(13)	O11-Cr1-O5	174.13(11)	O12^{i}-Ca1-O8	139.91(10)
N4–Cu1–N3	80.66(13)	O7-Cr1-O3	90.83(11)	O12i-Ca1-O6	88.11(10)
N4–Cu1–O2	95.90(12)	O7-Cr1-O1	172.46(11)	O12^{i}-Ca1-O6^{iii}	135.53(10)
N4–Cu1–O4	86.29(12)	O7-Cr1-O9	91.35(11)	O12^{i}-Ca1-O10^{i}	65.20(10)
N2-Cu1-O2	148.62(12)	O7-Cr1-O5	83.07(10)	O12^{i}-Ca1-O10^{ii}	74.42(10)
N2-Cu1-O4	81.94(11)	O3-Cr1-O1	82.49(11)	O8^{iii}-Ca1-O8	129.85(10)
N3-Cu1-O2	93.27(12)	O3-Cr1-O9	170.74(11)	O8^{iii}-Ca1-O6	78.40(10)
N3-Cu1-O4	159.10(11)	O3-Cr1-O5	90.14(11)	O8-Ca1-O6	66.99(10)
O2-Cu1-O4	71.81(11)	O1-Cr1-O9	95.80(11)	O8^{iii}-Ca1-O10^{i}	134.01(10)
		O1-Cr1-O5	93.40(11)	O8^{iii}-Ca1-O10^{ii}	84.47(10)
		O9-Cr1-O5	99.06(11)	O6-Ca1-O6^{iii}	91.42(9)
				O6-Ca1-O10^{i}	93.00(10)
				O6-Ca1-O10^{ii}	158.99(10)
				O10^{i} -Ca1-O10^{ii}	90.18(10)

Table S6. Thermoanalytical data for compounds **1**–**3.**

Comp.	Δ*t* / °C	*w* / %		Loss	*t*(DTA$_{max}$) / °C
		Exp.	Calcd.		
**1**	30–270	8.49	8.33	1.5H_2O	33 exo
	270–400	70.40	70.87	bpy, CO + CO_2	356 exo
**2**	35–220	9.99	10.37	2H_2O, 2CH_3OH, CH_2Cl_2	52 exo
	220–570	63.57	63.28	4bpy, 7(CO + CO_2)	236, 282, 307 exo
**3**	30–235	11.25	11.63	2H_2O, 4CH_3CN	48, 234 exo
	235–945	71.12	71.21	4phen, 6(CO + CO_2)	243, 305, 386 exo

Table S7. The best fitting parameters obtained from equivalent circuit modeling of complex impedance spectra measured at room temperature for compounds **1**–**3**, and calculated DC counductivity.

Compound	R / Ω	CPE		σ_{DC} / (Ω cm)$^{-1}$
		A / (s^a Ω^{-1})	a	
**1**	6.09×10^{11}	4.07×10^{-12}	0.87	2.21×10^{-12}
**2**	6.84×10^{10}	2.94×10^{-11}	0.63	1.33×10^{-11}
**3**	3.89×10^{11}	9.45×10^{-12}	0.74	2.41×10^{-12}

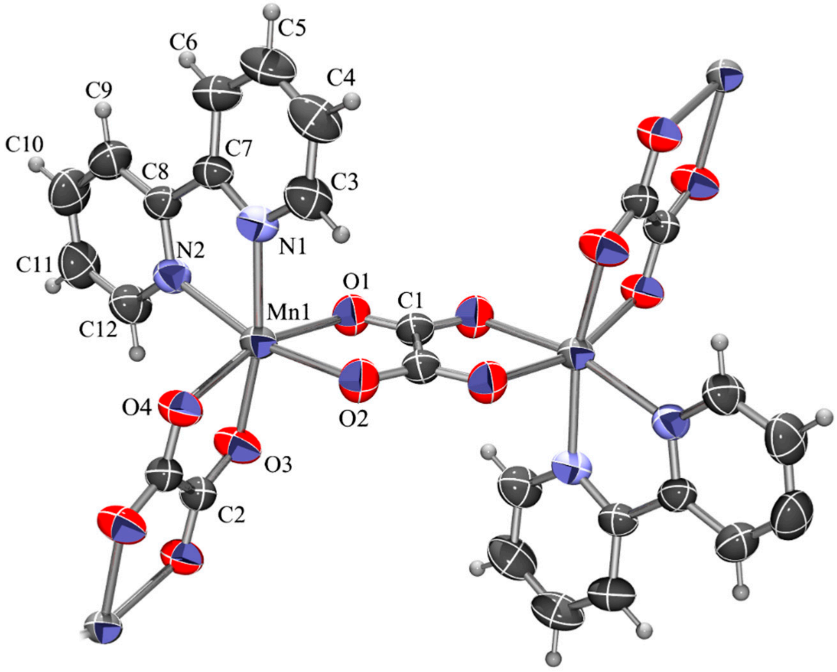

Figure S1. ORTEP-3 diagram of compound $\{[Mn(bpy)(C_2O_4)]\cdot1.5H_2O\}_n$ (**1**) with atom numbering scheme (only asymmetric unit is numbered). Displacement ellipsoids are drawn for the probability of 50% and hydrogen atoms are shown as spheres of arbitrary radii.

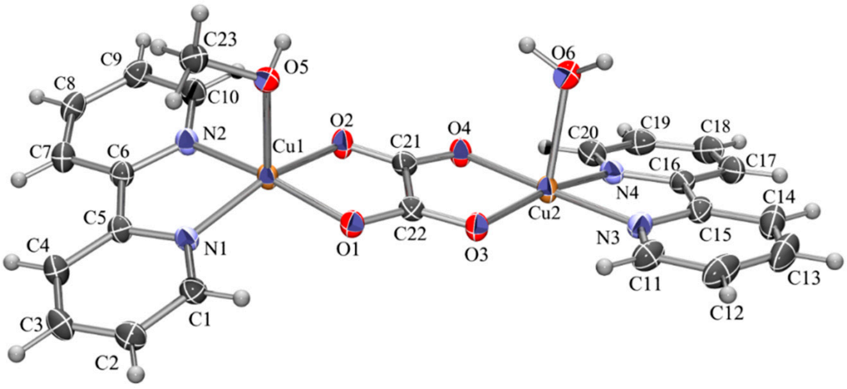

(a)

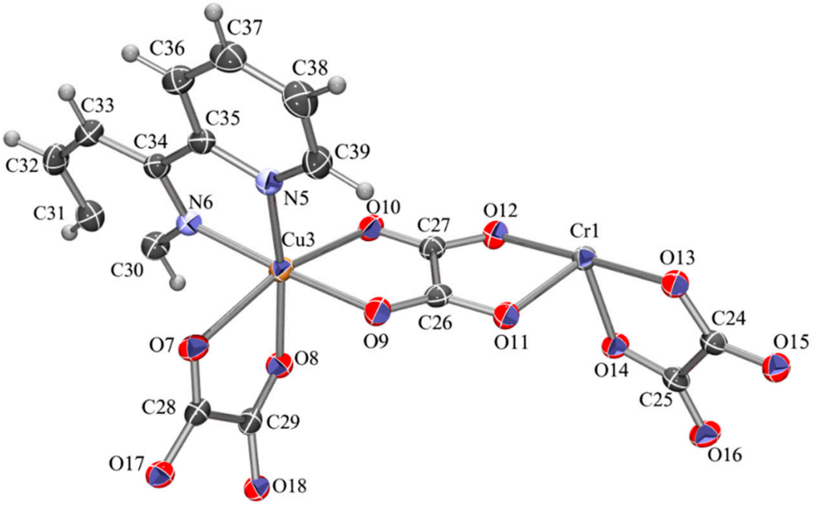

(b)

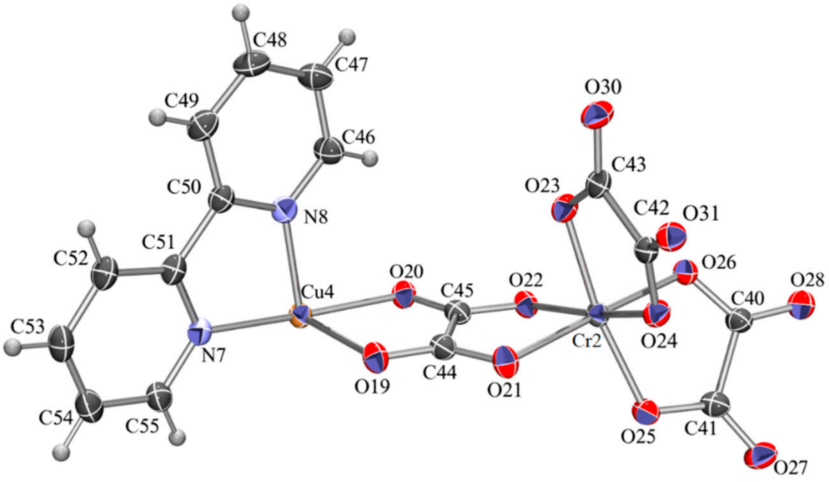

(c)

Figure S2. ORTEP-3 diagram of (a) dimeric unit **Cu1–Cu2**, (b) 1D chain **Cr1–Cu3** and (c) 1D chain **Cr2–Cu4** in compound **2** with atom numbering scheme. Displacement ellipsoids are drawn for the probability of 50% and hydrogen atoms are shown as spheres of arbitrary radii.

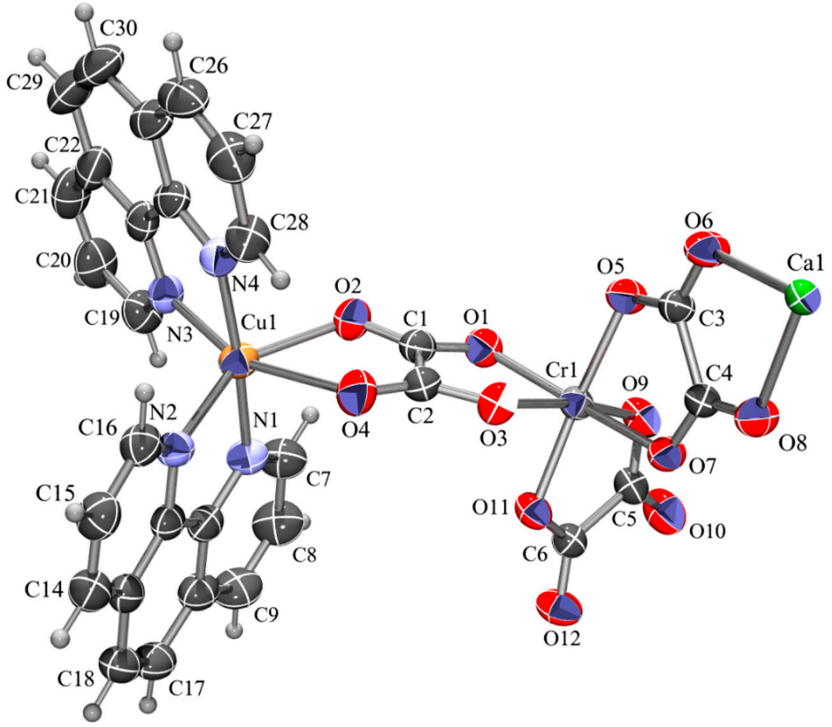

Figure S3. ORTEP-3 diagram of asymmetric unit of compound **3** with atom numbering scheme (uncoordinated water and acetonitrile molecules have been omitted for clarity). Displacement ellipsoids are drawn for the probability of 50% and hydrogen atoms are shown as spheres of arbitrary radii.

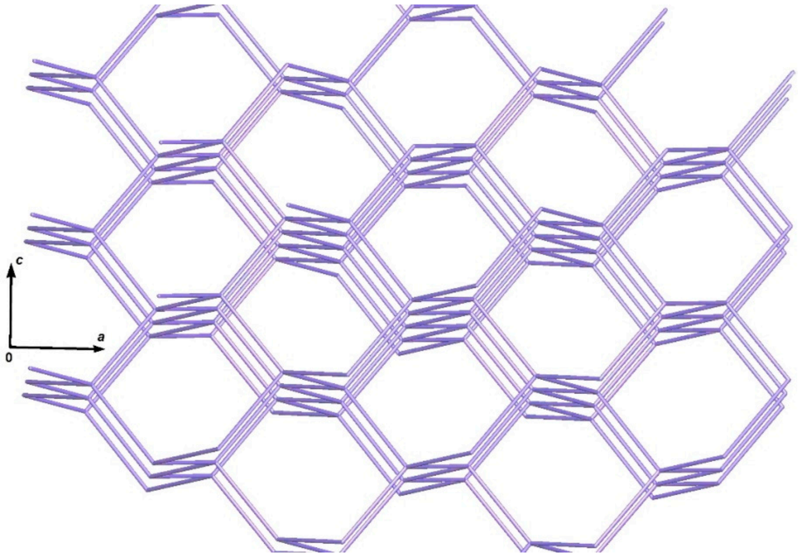

Figure S4. Underlying graph with **dia** topology in coordination polymer **3**. Nodes represent Ca atoms.

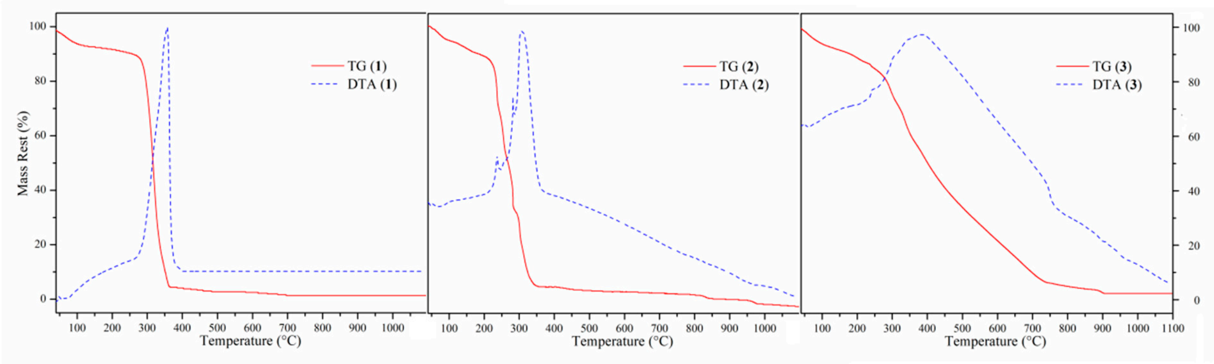

Figure S5. The TG and DTA curves for compounds **1–3** measured in nitrogen atmosphere.

© 2020 by the authors. Licensee MDPI, Basel, Switzerland. This article is an open access article distributed under the terms and conditions of the Creative Commons Attribution (CC BY) license (http://creativecommons.org/licenses/by/4.0/).
